# Irrigation of Young Olives Grown on Reclaimed Karst Soil Increases Fruit Size, Weight and Oil Yield and Balances the Sensory Oil Profile

**DOI:** 10.3390/foods11182923

**Published:** 2022-09-19

**Authors:** Maja Jukić Špika, Davor Romić, Mirella Žanetić, Monika Zovko, Tatjana Klepo, Frane Strikić, Slavko Perica

**Affiliations:** 1 Department of Applied Sciences, Institute for Adriatic Crops and Karst Reclamation, Put Duilova 11, 21000 Split, Croatia; 2 Centre of Excellence for Biodiversity and Molecular Plant Breeding, Svetošimunska 25, 10000 Zagreb, Croatia; 3 Department of Soil Amelioration, University of Zagreb Faculty of Agriculture, Svetošimunska 25, 10000 Zagreb, Croatia; 4 Department of Plant Sciences, Institute for Adriatic Crops and Karst Reclamation, Put Duilova 11, 21000 Split, Croatia; 5 Center of Pomology, Croatian Agency for Agriculture and Food, Kralja Zvonimira 14a, 21210 Solin, Croatia; 6 Department of Marine Studies, University of Split, R. Boškovića 37, 21000 Split, Croatia

**Keywords:** virgin olive oil, irrigation, young trees, sensory analyses, phenols, fatty acid profile

## Abstract

The influence of different irrigation regimes on olive fruit morphological parameters and on the quantity and quality (marketable indices, phenolic content, fatty acid composition, and sensory profile) of virgin olive oil (VOO) obtained from the Croatian cultivar Oblica, grown on an extremely rocky and dry reclaimed karst soil, was studied over three years. Four treatments were applied: rain-fed and three treatments calculated as 50%, 75%, and 100% of the crop’s irrigation requirement (Irr). Principal component analysis separated growing seasons (GS) that differed in precipitation. In the 2016 season, which had a low number of fruits per kilogram and provided a higher amount of balanced VOO with medium to intense bitterness and pungency (rain-fed treatment), the oil yield increased by irrigation (Irr 75 and Irr 100) up to 18%, while unchanged phenolics, bitterness, and pungency were observed for the VOOs obtained. In the drier GS (2017), which under rain-fed conditions had high fruit per kg, smallest fruit sizes, and lowest oil yield, and in which the VOOs had high phenolic content and intense sensory taste attributes, fruit weight, fruit sizes, and oil yield increased by 35% in all irrigation treatments, while phenols, bitterness, and pungency decreased, balancing the sensory profile of the VOOs. The results obtained here led us to conclude that the irrigation of young olives resulted in a positive effect, with the indication that an abundant water supply is more effective in drought conditions.

## 1. Introduction

Irrigation may be the single most strategically important intentional environmental modification humans have learned to perform [1]. The driving force motivating farmers and governments in regard to irrigation is increased production, higher incomes, direct and indirect employment, and contribution to GDP [2]. Although olive trees are considered to be quite drought resistant [3], olive cultivation in Southern Europe stopped relying on rain-fed agriculture a long time ago, and the positive response of olive trees to irrigation is well known [4,5]. In addition, our interest in irrigation is triggered by the new challenges arising from climate change that threatens this traditional crop. The Mediterranean region is considered a climate change “hotspot”, already facing significant warming and drying trends and expected to do so in the future. In this context, climate change may become a particular challenge for olive growers [4]. 

Croatia followed the trend of introducing irrigation in the olive sector only a decade or two ago. Our traditional olive groves, where mainly the local Oblica variety is grown, were able to survive the hottest and least rainy summers and bear some fruit. The increasing interest of our growers in olive irrigation coincides with the trend of planting olives on reclaimed karst land, land that was brought into agricultural production for the first time. The area under olive cultivation in the Republic of Croatia increased by 60% in the period 2000–2014, but expectations of higher olive yields have not been met. The main reason for this is that many of the newly planted olive orchards have not yet come into full production. We assume that the reason is a delay in the growth and development of many newly planted olive groves because they were mostly planted on reclaimed karst. Actually, reclaimed karst, the land where new olive orchards are mostly established, is a very marginal land obtained from limestone by ripping, crushing and grinding. Such land is known for its high content of rock fragments and weak physical properties. The massive planting of new olive groves on the reclaimed karst soil has occurred rapidly and without sufficient knowledge of the soil itself, as well as without sufficient comprehensive and specific knowledge of olive cultivation on such soils. 

Increasing productivity while maintaining the highest possible oil quality is the main goal of all agrotechnical and technological developments. Considering that fruit quality is important for the table olive and olive oil industries, morphological analysis can be an effective tool for characterizing and distinguishing cultivars, forming associations between them [6], but also between different agro-technical practices. The application of organ-level dimensional phenotyping to describe morphological parameters is of great importance to plant phenomics research and is usually part of phenotypic data collection as a basis for the multiple analyses that can be subsequently performed [7]. Several studies on olive trees grown under different water regimes have shown that fruit morphological characteristics, such as longitudinal and equatorial diameter, volume, and pulp:stone ratio, can improve [8,9,10]. Olive oil yield most closely reflects olive fruit growth and development, which in turn depends on the optimal availability of water [4,5]. Irrigation increases the water content of the mesocarp [11], which negatively affects the amount of oil separated [12]. It should be noted that dry matter and oil content are cultivar dependent traits [13], so the changes that occur either agrotechnically or later during oil processing can hardly be evaluated as a definite rule. For the VOOs obtained, the market quality parameters, e.g., free fatty acids, peroxide value, and spectrophotometric indices in the ultraviolet range, most authors agree that the application of different irrigation strategies has little or no effect [5,10]. However, in several previous studies, a negative correlation was observed between the amount of water applied to olive trees and phenolic content, the primary antioxidant of VOO [5,14]. Enzyme activity has been hypothesized to be higher under stress conditions [15], although results have been found supporting the opposite effect of irrigation on phenolic compounds [16], as cultivars have different abilities to adapt to changing conditions of temperature and available water [16,17]. Changes in phenolic content directly affect the oxidative stability of the oils, but also the sensory characteristics, which are an important distinguishing feature of VOOs compared to other vegetable oils. The response of different influencing factors, and in this case the effect of irrigation, in an olive and consequently in a VOO in which several compounds have interactive, synergistic, or antagonistic effects could be observed by looking for differences in these key properties of the oil, which combine odor, taste, and retro-nasal properties.

Thus, the purpose of this study was to provide insight into the development of a water-efficient management strategy for an olive orchard established on stony soils in an area with low rainfall and frequent occurrence of high air temperatures during the growing season. The specific objectives were to evaluate the impact of different irrigation regimes based on olive water use quantification (50%, 75%, and 100% of crop evapotranspiration), which was estimated by measuring (i) fruit and stone morphological parameters (weight, width, length, area, aspect ratio, perimeter, shape, volume), (ii) fruit and oil yield, and (iii) virgin olive oil quality (acidity, peroxide value, specific extinction coefficient at 232 and 270 nm, phenolic content, fatty acid composition, and sensory profile) obtained from young trees grown on extremely rocky and dry reclaimed Mediterranean karst soils in three years with different duration and intensity of high temperature periods during irrigation.

## 2. Materials and Methods

### 2.1. Site Characteristics and Environmental Conditions

The study was carried out from 2015 to 2017 in the commercial olive orchard (*O. europea* L. cv. Oblica) located in Jadrtovac (Šibenik County, Croatia) at 90 m altitude, 5500 m distanced from the sea, and positioned at 43°39′54″ N, 15°58′45″ E. The orchard was established in 2010 on the reclaimed karst soil that was described in detail by Romić et al. [18]. Briefly, the olive orchard soil was obtained from a shallow rocky brown soil on limestone by ripping it up to 60 cm in depth followed by the stone crushing and grinding the surface layer to 25 cm depth. The obtained soil is notorious for its high proportion of rock fragments, small ratio of fine soil particles, and low water holding capacity. From planting, until the irrigation experiment was established, the trees were dry farmed. At the beginning of the experiment, the average five-year-old tree’s height was 184.54 cm, tree width (N–S and E–W) 207.0 cm and 209.8 and cm, trunk diameter (N–S and E–W) 72.0 cm and 72.5 cm, respectively. 

The orchard is influenced by the Mediterranean climate, defined as the Csa climate type [19] and the Walter–Lieth climate diagram [20] is presented in Figure S1 (data obtained from the Meteorological and Hydrological Service of Croatia). The weather is characterized by hot dry summers and mild rainy winters. The maximum accumulated rainfall is during November with at least three times as much precipitation in the wettest month of winter as in the driest month of summer. The area is generally windy, with an average annual wind speed of 3.9 m s^−1^. All climatic parameters, e.g., radiation, insolation, and evapotranspiration, reach their maximum in July, except for wind speed. 

**Figure 1:**
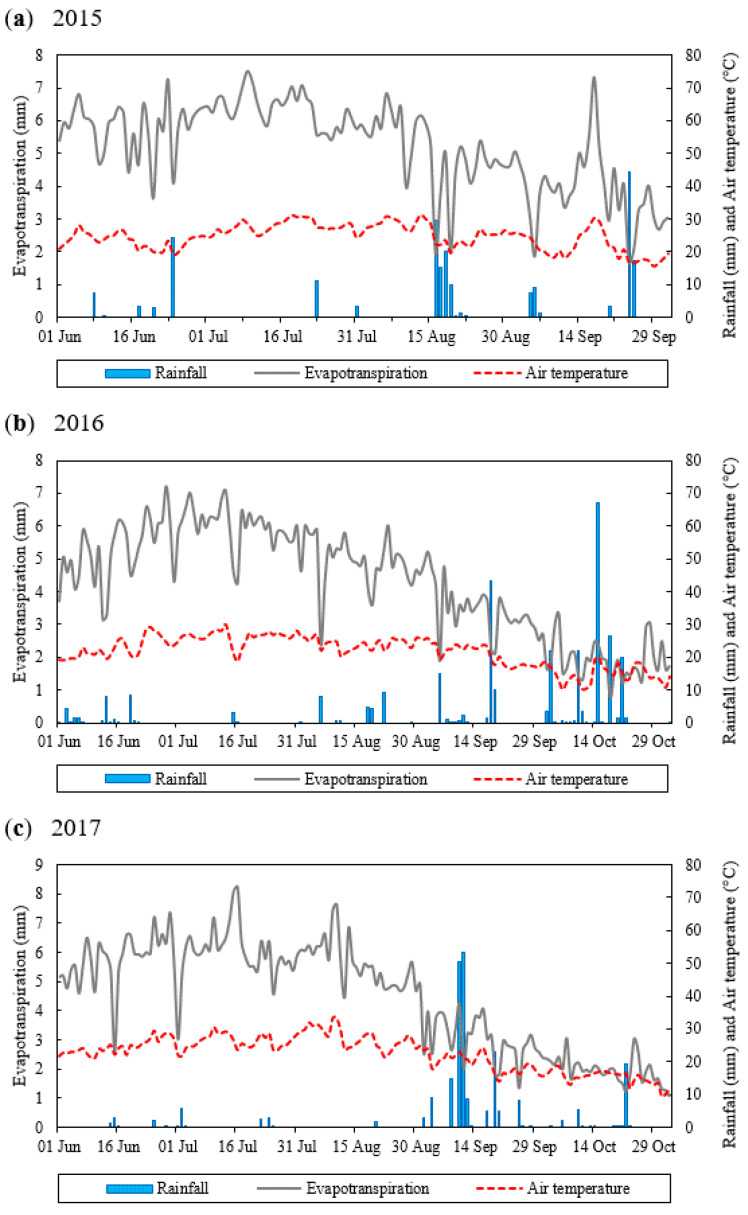
Rainfall, air temperature and evapotranspiration during the growing seasons of (**a**) 2015, (**b**) 2016 and (**c**) 2017.

Due to the significant variation in specific climatic features over short distances, data collected from the Šibenik meteorological station were not considered completely reliable for the study site. Therefore, during the period under study (Figure 1), an automated weather station was installed at the study site (Pinova Meteo Weather Station http://pinova-meteo.com/hr_HR/, accessed from 1 January 2015 till 31 December 2017) to record the air temperature, relative air humidity, precipitation, leaf moisture, wind speed and direction, solar radiation, and soil moisture, in 10-min intervals.

Annual precipitation values were 1015.7 mm, 757.1 mm, and 769.3 mm, respectively, for 2015, 2016, and year 2017 (Figure S1). The total annual rainfall in 2015 was considerably higher whilst in 2016 and 2017 was close to the average long-term precipitation average (765.55 mm). In the period of olive pit hardening to the stage of full fruit development (correspond to June till August), total precipitation amounted to 129.4 mm, 57.7 mm. and 19.7 mm for the years 2015, 2016, and 2017, respectively. In the same period, temperatures were similar, except in August, in which recorded temperatures were higher in 2017 compared to the other two years (Figure 1 and Figure S1). 

The reference evapotranspiration (ET_0_) from Pinova Meteo Weather Station and crop coefficient were used for irrigation management. Irrigation amounts were enough to replace the crop evapotranspiration estimated with the crop coefficient approach [21]. The crop coefficient values were those estimated for the olive orchard by [22]

### 2.2. Experimental Set-Up

In July 2014, a reconnaissance of the terrain was performed in the olive grove consisting of the five-year-old autochthonous predominant Croatian cultivar Oblica. The trees were planted at a spacing of 6 × 7 m. The irrigation experiment was set up as a randomized complete block design with four levels of irrigation in five replicates. The single experimental plot (replicate) consisted of three olive trees surrounded by guard trees to avoid interferences among treatments. The four treatments started in 2014 and were applied for four years (Table 1). 

Based on the daily water balance, drip irrigation was applied. Evapotranspiration lost on rainless days was compensated by irrigation, while on rainy days irrigation was postponed until the sum of effective precipitation had been consumed by ETc. To the main irrigation line placed along each tree row, a smaller pipe was attached to surround each experimental tree separately (covering olive tree canopy), which was equipped with drippers of appropriate capacity to ensure water delivery according to the experimental design and irrigation treatments so that the same irrigation time is kept for all variants of the experiment (Table 1). The web-based GALCON GSI irrigation controller (http://galconc.com/, accessed from 1 January 2015 till 31 December 2017) was used for remote irrigation system management. The orchard was organically managed, with the same management practices applied for all experimental plots. Heavy pruning was performed in the first year of the experiment.

### 2.3. Olive Fruit Sampling

Olive harvest was performed in 2015, 2016, and 2017 when fruits had the ripeness index of cca. 2.5–3 (determined based on skin color and pulp [23], precisely on 1 October 2015, 2 October 2016, and 10 October 2017. In 2015, the fruit yield was low for fruiting per tree and the samples were averaged by harvesting and combining fruits from all trees used in a particular treatment, after which only chemical analyzes were performed. In the other two growing seasons (2016 and 2017), approximately 500 g of healthy olives per tree were sampled along four transects (SE, NE, SW, and NW) and used for morphological measurements. For olive processing and subsequent analysis, an additional 2 kg per tree of fruits were hand-harvested. In both cases, the yield per tree was recorded, after which the fruits from the three trees that make up each of the repetitions were merged for oil processing and further morphologic analyses. To calculate the fruit yield per tree, all the remaining fruits were mechanically harvested and weighted. The obtained mass per tree was summed up with the fruit masses used for morphological measurements and the oil production.

### 2.4. Olive Fruit and Stone Morphologic Analyses

Forty fruits per repetition were randomly selected from the homogenized batch contained samples harvested from three trees and weighted with a precision up to 0.01 g. On the same fruits, image analysis (quantitative morphological characterization) was performed using Win FOLIA Pro software (Regent Instruments Inc., Quebec City, QC, Canada). After the separation of the pulp from stone, the stone weight and image analysis of the stones were performed. Quantitative morphological characterization, except for the weight, included: area as the area inside the polygonal line-contour (A), horizontal width (Width; HW), vertical length (Length; WL), aspect ratio (Aspect Ratio (W/L); AR), perimeter as the total length of the polygonal line-contour (P) and shape (S). For both fruit and stones, the shape was calculated as the relation between horizontal width and vertical length. The volume of fruits and stones was estimated using the formula:

$$V=\frac{4}{3}\;\pi \;\frac{\mathrm{vertical}\;\mathrm{length}\;}{2}\;x\;{\left(\frac{\mathrm{horizontal}\;\mathrm{width}}{2}\right)}^{2}$$

### 2.5. Oil Extraction and Oil Yield

Olives were processed using laboratory oil mill Abencor (mc2, Ingenierias y Sistemas, Sevilla, Spain), which simulates the industrial process of VOO production. Each sample was obtained separately, and the mill parts were cleaned in between. Processing parameters have been described in a previous paper by Jukić Špika et al. [24]. Olive fruits were milled in the hammer crusher, and then the olive paste was kneaded for 35 min at 26 ± 2 °C in the thermal beater. After the vertical centrifugation of 1370 g for 70 s, the oily must was collected and left to decant. Oil samples were stored in darkness without headspace and at 18 ± 2 °C until analysis. Oil yield was calculated as the ratio of the mass of extracted oil and mass of olive paste used for the extraction, and expressed as a percentage of fresh weight, considering oil density at the ambient temperature of 0.916 kg L^−1^.

### 2.6. Analyses of Quality Parameters of Olive Oil and Sensory Evaluation

Determinations of free fatty acids (FFA), peroxide value (PV) and spectrophotometric indices (K_232_, K_270_ and ∆K) were made following the analytical methods described in the EC Regulation [25]. The sensory evaluation was conducted according to the International Olive Council [26] methodology where odor and taste attributes were quantified using a 10-cm unstructured intensity ordinal rating scale from 0 (no perception) to 10 (the highest intensity). The panel consisted of eight experienced and well-trained experts in VOO tasting. Encrypted olive oil samples (15 g) were presented to the evaluators in covered blue glass previously heated to a temperature of 28 ± 2 °C. 

### 2.7. Analyses of Phenols

The content of phenols (TPC) was determined using the colorimetric method described by Gutfinger [27]. Total phenols were isolated by liquid-liquid extraction of a solution of oil in hexane with a water/methanol mixture (60:40, *w*/*w*), three times. The colorimetric reaction was performed using Folin-Ciocalteu reagent (Sigma-Aldrich, St. Louis, MI, USA), and the determination of the phenol was measured at 765 nm on a Cary 50 spectrophotometer, UV-VIS (Varian, California, USA). Results were expressed as mg of gallic acid per kg of oil.

### 2.8. Analyses of Fatty Acid Composition

Determination of fatty acid composition [28] was carried out by gas chromatography, using an Agilent 6890N GC System (Santa Clara, CA, USA) equipped with a flame ionization detector (FID), with the prior esterification of fatty acids that were made according to ISO [29]. The methyl esters were separated on a DB-WAX column (30 m × 0.25 mm × 0.25 μm). Helium was used as carrier gas with a flow rate of 1.5 mL/min. Injector temperature was set at 250 °C and detector temperature at 280 °C. The oven temperature was programmed to rise 7 °C/min from 60 °C to a final temperature of 220 °C, holding an additional 17 min. The split ratio was set to 30:1. Based on retention times of the standard mixture of fatty acids and methyl esters, the identification of particular fatty acids was performed. Calculation of the quantitative composition of fatty acids was carried out by means of the normalization surface method. Results for fatty acid composition were expressed as the percentage of total fatty acids present in olive oil. The percentage of total saturated (SFA), monounsaturated (MUFA), and polyunsaturated (PUFA) fatty acids was calculated. 

### 2.9. Statistical Analysis

All variables were examined separately by analysis of variance (ANOVA) and means separations were performed by Tuckey tests at *p* ≤ 0.05, to determine significant differences among the applied irrigation treatments. To conclude and summarize, the multivariate analysis of all data from 2016 and 2017 (years in which all morphological and chemical analyses were conducted) was performed and the results are presented in the selected two-factor PCA model. All statistical analyses were performed using Statistica 14.0.0.15 (Tibco Software Inc., Palo Alto, CA, USA, 2020)

## 3. Results and Discussion

### 3.1. Influence of Irrigation and Growing Season on Fruit Morphology, Fruit and Oil Yield

Irrigation treatments (as the main factor) affected the morphological parameters of olive fruits (Table 2). Fruit area was highest in fully irrigated trees (Irr 100; 4.07 ± 0.74) and showed a gradual significant decrease with the decrease in the amount of water received, and finally fruits from non-irrigated trees had the lowest area (C; 2.55 ± 0.96). The same pattern was observed for the average width, length, perimeter and volume of the fruits, while the aspect ratio of the fruits (W/L) and the shape coefficient decreased with irrigation. The fruits of the studied cultivar Oblica obtained in this study can be described as oval, with an average width of 1.93 cm and length of 2.14 cm (Table 2). These values are similar to those obtained when describing the fruits of Istarska bjelica, while they are larger than the fruits of Leccino [30]. Although these parameters have been shown to be cultivar dependent [30], our results on the influence of irrigation on fruit size parameters are in agreement with previously published results [8,31]. 

**Figure 2:**
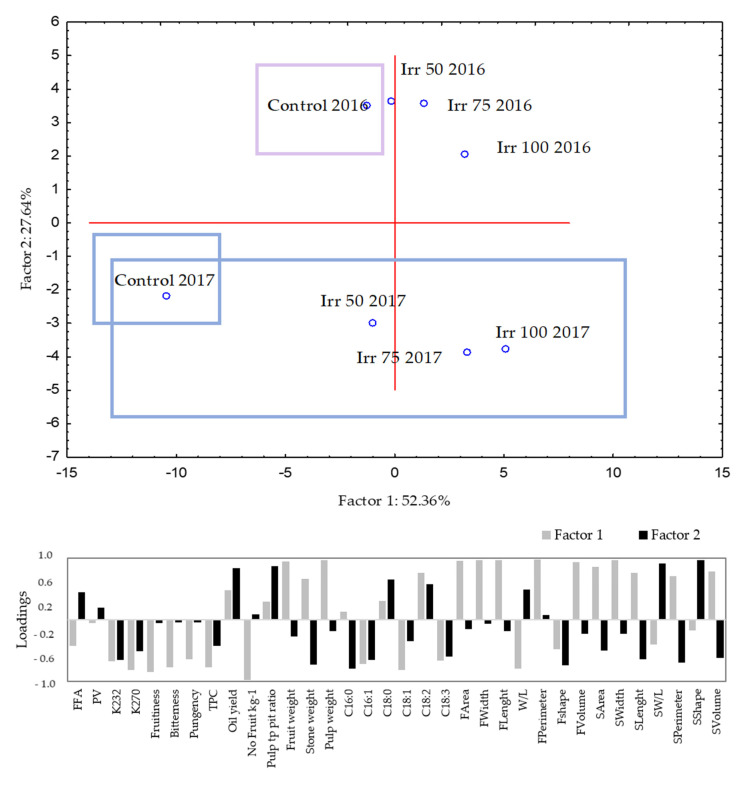
Observation plot of the principal component analysis (PC1 vs. PC2) showing the dispersion of samples on the measured morphological, chemical and sensorial parameters; and the diagram of the loadings for the analyzed parameters.

In addition, the growing season showed a significant influence on some of the olive fruit characteristics measured (width, W/L, perimeter and shape), while an interaction between irrigation treatment and season was found for all parameters (Table 2). From the observation diagram of principal component analysis (Figure 2), it can be seen that irrigation treatments in two analyzed growing seasons influenced the measured parameters to different degrees. It can be seen that irrigation had a greater impact in 2017 (the year with less rainfall during pit hardening and the intense growth phase of the fruit compared to 2016) (Figure 1), where even with the lowest amount of water applied, 50% ET, significant differences were observed. An increase in the width, length and volume of the fruit was obtained. In 2016, the increase compared to rain-fed olives was achieved only with full irrigation (Ir 100).

As for the diameters and shapes of the stones (Table 3), the maximum longitudinal and equatorial diameters of the stones corresponded to those obtained with the highest amount of water applied (Irr 100; 1.4 ± 0.18). The same changes due to irrigation were observed for stone area, width, perimeter, and volume (Table 3). Morales-Sillero et al. [31], in their localized irrigation experiment conducted in pots and in the field over two years, found changes that were greater in the irrigation treatment than in the rain-fed treatment only in the second year of the study. The growing season also had a significant effect for all variables measured for the stones (Table 2), and the stones from 2017 were larger and more elliptical (lower shape coefficient) than those from the 2016 season. An interaction between irrigation treatment and season was found for all parameters, and the effect of irrigation on the morphological characteristics of the stones was less evident in 2016, where only the Irr 100 treatment resulted in a significant increase in the measurements (Table 2, Figure 2).

Fruit yield per tree was influenced by irrigation regimes, but variability was observed that was not clearly related to the amount of water applied between irrigation treatments (Table 4). The growing season and the interactive effect of the main factors were also recorded. The year 2016 had a higher fruit yield, but after analyzing the data, it is obvious that there were no differences between treatments. In 2017, the lowest yield was recorded in the control treatment (rain-fed), and it differed only compared to Irr 75, which had the highest yield (Table 4). It is well known that olive is well adapted to drought and semi-arid conditions, but increasing productivity is a very important commercial objective. The results of irrigation treatments were variable, in cv. Picual no significant differences were found between different irrigation treatments [32], in Leccino trees receiving 50% of their water requirement the yield was 19% lower than in fully irrigated trees [11], in rain-fed Leccino trees the fruit yield was 35% lower than in fully irrigated trees [33]. The differences result from the different cultivars studied, the characteristics of irrigation (constant or in specific periods), alternative bearing, vegetative growth, reproductive growth, and orchard density [32,34,35,36,37], but also from the type of soil. The present study was conducted on low-growing trees (184.54 cm, 207.00 cm, and 72.03 cm; tree height, width, and trunk diameter, respectively) that were heavily pruned in 2014 and grew on soils with low water retention capacity and low available soil moisture. Part of the yield difference can be attributed to smaller fruit size (Table 2), but trees likely need more time for more vigorous growth and a resulting increase in yield, with or without applied irrigation.

The number of fruits per kilogram was highest in the non-irrigated treatment, and no significant differences were observed compared to the treatment with the least irrigation (Irr 50) (Table 4). As irrigation increased, the number of fruit per kilogram decreased, and the treatment with the most accessible water (Irr 100) had the lowest number per kilogram but also the highest fruit weight. The variations in fruit weight per treatment are interesting. The differences in fruit weight were not proportional, and the largest difference was found between the control treatment (C) and the treatment with the lowest irrigation (Irr 50) (difference = 0.93 g). There were no significant differences between the two years, although there was an interactive effect for irrigation × season, and in 2016 higher fruit mass was obtained only with Irr 100, while in 2017 significant changes were already obtained by the Irr 50 treatment. Although pulp to pit ratio was generally not affected by the treatments applied, when we consider the effects of the main factor, individual effects were observed in each year (Table 4). Since Oblica is a dual-purpose cultivar, its weight and size parameters, apart from oil production, are also of particular importance for the table olive industry. Even considering fruit quality from this point of view, our results show positive effects of irrigation (especially in Irr 75 and Irr 100 treatments) on obtaining adequate fruit weight and suitable size parameters (pulp to stone ratio, volume, width and length) (Table 2, Table 3 and Table 4). The improvement of these morphological parameters by irrigation treatments is consistent with previous results [11,31,38].

Irrigation as the main factor affected oil accumulation in fruits (F = 12.413, *p* ≤ 0.0001). By Irr 50 (12.76%), oil yield was about 20% higher than in the control treatment (C; rain-fed; 10.25%). At the same time, oil yield of Irr 50 was more than 90% (93.5%) that of fully irrigated trees (Irr 100) among which there was no statistically significant difference (Table 4). Thus, irrigation even at Irr 50 allowed us to maintain the oil yield of fully irrigated trees, thus saving water. An increase in oil content was reported by Dabbou et al. [39], who also set a linear irrigation experiment on cv. Arbequina in which trees received a fixed percentage of their evapotranspiration requirement during the irrigation period. Morales-Sillero et al. [40], who studied the cultivar ‘Manzanilla de Sevilla’ under localized irrigation and non-limiting soil water conditions, reported the opposite, a decrease in oil yield with irrigation compared to the rain-fed treatment. In most cases, irrigation had no effect on oil content expressed as fresh or dry weight [41,42]. The type of soil, but also the resistance of the cultivar to drought, most likely contribute to the differences in the results of the different studies on the effects of irrigation on oil yield. In our previous study on rain-fed Oblica grown on clay loam soils with alkaline reaction and low to medium skeleton content, oil yield ranged from 10.3% to 17.7% with an average fruit mass of 5.72 g (3.16–7.98 g) and extractability of 67.3% (41.2 to 87.3%) [43]. A negative role of irrigation has been frequently reported for physical oil extraction [44,45,46], as an inverse relationship between fruit water content and oil extractability [12] has also been reported for Oblica [43]. Oblica is usually considered a drought-resistant cultivar. However, in the present study, the fruits of the control treatment were small and had significantly lower weight. Thus, irrigation allowed normal growth and flesh development, resulting in a weight close to the usual values for Oblica in treatments Irr 75 and Irr 100, and finally, a higher oil yield was obtained in these treatments compared to rain-fed (control) (Table 4).

The growing season, as the main factor, also had a strong Influence on oil yield. In 2016, the yield was significantly higher than in 2017, and in the case of the control, the difference was more than twice as large (Table 4). In each season, irrigation resulted in a positive effect; in 2016, which generally (as an average of all treatments) had a low number of fruits per kilogram and smaller fruits with lower weight, oil yield increased by up to 18% due to irrigation (Irr 75 and Irr 100). Calculating the yield per tree, the total oil yield was 37 and 47% higher for Irr 75 and Irr 100, respectively, compared to the control treatment. On the other hand, 2017 had a higher number of fruits per kg, and as an average of all treatments the fruits were larger and had a higher weight. In this year, the irrigation treatment resulted in a higher oil yield of about 35% (40–38–33%; Irr 50–Irr 75–Irr 100). More accessible water did not significantly increase oil yield. One possible reason for the significantly lower oil yield in 2017 was a very dry period that year during the pit hardening and intense fruit growth (Figure 1; corresponding to June, July, and August), which resulted in slower fruit growth. Although overall oil yield was lower in 2017 (as an average of all treatments), this year, as mentioned earlier, a greater shift was achieved compared to 2016 (a 35% increase compared to 18%; 2017 and 2016) (Table 4). This is consistent with the hypothesis of Caruso et al. [10] that abundant water supply is more effective in dry/warm years. Several irrigation studies have reported that differences between growing seasons may also be due to differences in fruit yield, i.e., alternative bearing [11,31], to which Oblica is extremely prone. However, in this experiment, young trees were used in which this trait should be less pronounced. The fruit yield data obtained also do not support this theory (Table 2), which is consistent with Ben-Gal et al. [47], who studied the influence of storage cycles on olive oil production in response to irrigation.

### 3.2. Influence of Irrigation and Growing Season on Quality, Phenols and Sensory Characteristics

All olive oil samples obtained (Table 5) were classified as “extra virgin olive oil” according to EU Regulation 2568/91 [25], indicating good fruit quality and the absence of higher hydrolytic and oxidative oil alterations. Free fatty acids ranged from 0.30% to 0.50% oleic acid, with the lowest free fatty acids found in oils from olives receiving most water (100% ET). The peroxide value (3.86 to 6.86 meq O_2_ kg^−1^) was not affected by irrigation. The specific absorbances in the UV part of the spectrum (K_232_, K_270_) were significantly lower in oils from irrigated trees (Table 5). However, the effects of the main factors interacted significantly, and lower K numbers were observed between treatments in 2017, while no specific response of K numbers to irrigation was visible in 2015, and irrigation treatment did not cause significant changes in 2016 (Table 5). Although significant changes in quality parameters are visible due to irrigation and growing season, the changes are small and no clear trend can be shown. Different results were also obtained for the physicochemical parameters evaluated in other irrigation studies [31,38,48,49,50].

The results of the two-way analysis ANOVA show significant differences in phenolic content between irrigation treatments and between growing seasons (Table 5). The concentration of phenols decreases with irrigation, which is consistent with previous findings suggesting that water deficit can lead to increased synthesis of phenolic compounds in olives and associated VOOs [51]. The increased synthesis was likely due to changes in enzyme activity involved in synthesis of secoiridoid compounds as main phenolic compounds in olive fruit and VOO. The key enzyme for the hydrolysis of fruit phenolic glycosides during olive oil extraction is endogenous β-glucosidase, and changes in its activity directly affect the final phenolic content and phenolic profile in VOO [52,53]. Consequently, trees that received a higher amount of water probably yielded oils with lower phenolic concentrations (Table 5).

However, in the present study, the main effect of growing season shows higher phenol variability (F-statistic value) than irrigation, which was highest on average in 2017 (Table 5). The results are consistent with the literature, namely in that the variability of oil phenols within crops is influenced by the growing season [54,55] as well as other agronomic and technological factors [56].

There was a significant interaction between the main factors (irrigation × growing season) (F = 13.69, *p* ≤ 0.0001) (Table 5). Irrigation resulted in significant differences in 2015 and 2017, while no differences were observed between treatments in 2016. In 2015, the TPC was lowest at 75% ET, but still 80% of the TPC of the oils from the non-irrigated treatment (control). Irrigation had a significant effect in 2017 when TPC decreased linearly (y = −225.78x + 1373.3; R² = 0.9287), and finally, phenolics were 65% lower in oils from fully irrigated trees (100% ET) than in the control treatment (Table 5). In contrast to the results of Servili et al. [38], the VOOs of non-irrigated trees differed strongly between harvest periods, with particularly high phenolic concentrations in 2017 (C in 2015—641.93 ± 10.24; C in 2016—569.23 ± 148.44; C in 2017; 1099.94 ± 152.23 mg kg^−1^) (Table 5). The data on olive fruit parameters from the 2017 control treatment (Table 2 and Table 4) show that the fruits were quite small, had the lowest pulp to pit ratio and yield only 6.4 ± 0.09% oil. Although irrigation in this year also reduced phenol concentration, it allowed us to have normal fruit growth and development (Table 2 and Table 3) and lipogenesis (Table 4). Importantly, the treatment with the lowest water addition (Irr 50; 940.38 ± 124.91 mg kg^−1^) did not differ from the control treatment in terms of phenolics (Table 5) and can be considered as VOOs with high phenolic content [57]. The VOOs of Irr 75 (801.34 ± 109.82 mg kg^−1^) fell into the same category. This is important for the classification of olive oil as a “health-promoting food” [58], since oils containing more than 500 mg kg^−1^ should be able to safely comply with the health claim limit for at least 12 months after bottling [57]. 

Phenolic components, which are thought to be largely responsible for the beneficial effects of VOOs on human health [59,60], are positively related to the extension of the shelf life of the oils and are also closely related to the organoleptic properties of VOOs [61,62]. They give VOOs their unique flavor in the form of bitterness and the chemical-esthetic sensitivities of pungency and astringency. Considering this, it is understandable that irrigation very often affects the sensory properties as it does the phenolic compounds, or at least shows the same pattern of changes. No defects were found in the olive oil samples obtained for this study, and according to the sensory evaluation rules [63], all oils were classified as “extra virgin olive oil” (Table 5). The results discussed in this paper indicate significantly different sensory profiles of the VOOs with respect to the applied irrigation treatments (main factor). In general, these oils were of medium fruitiness, with bitter and pungent attributes of high intensity. Although the differences were statistically confirmed, the direction of change with respect to the fruity and pungent taste attributes is not clear. Bitterness differed between the control and Irr 100 (the treatment with the highest amount of water), where the least bitter oils were evaluated (Table 5). Oblica oils are described as having a harmonious medium fruitiness and flavor with medium bitterness and medium to intense pungency [24,64], although Oblica oils were found to have intense bitterness and pungency and slight astringency at the beginning of the harvest season (green olives) or were from the colder growing area at higher altitudes [24]. 

A significant interaction for monitored sensory traits was observed between the main factors (irrigation × growing season) (Table 5). In 2015 and 2016, the intensities of taste sensory traits were similar for all applied treatments. This can be considered an encouraging result, since it was possible to improve fruit morphological parameters and oil yield without negatively affecting sensory quality. The year 2015 was significantly rainier compared to the long-term precipitation mean (Figure 1 and Figure S1) and to two other years studied, so irrigation was probably not as significant, i.e., the differences in the amount of water supplied between treatments were sufficient to reflect the changes in the sensory characteristics observed. Comparing 2016 and 2017, they had similar rainfall values on an annual basis, but they differed significantly in rainfall during the period of olive pit hardening and full fruit development. 2017 was a year of extremely low rainfall (577 mm and 197 mm for 2016 and 2017, respectively; Figure 1). Thus, 2017 proved to be the year with the greatest significant differences in sensory characteristics (Table 5). PCA analyses confirm these, where it can be seen that the control sample in 2017 (rain-fed treatment) was particularly disjointed, where high negative correlation of Factor 1 was recorded with the frutiness, bitterness, and pungency (Figure 2). Fruitiness and pungency differed significantly, with lower values in the Irr100 treatment. A decrease in fruitiness with irrigation was also observed for the cultivar cv. Coratina grown on karst soils, which was associated with a decrease in E-2 hexenal and C6 volatiles [65]. In the cultivars Arbequina, Coratina, Koroneiki, and Cobrançosa, the oils from rain-fed, less irrigated, or deficit irrigated (30% ETc) were more pronounced than the oils from fully irrigated trees [66,67,68]. The different responses of the same cultivar to the same irrigation treatment but applied in different growing seasons were reported by Fragepane et al. [5]. In the present study, the intensity of the pungency of the control treatments between years is interesting because it was one unit of measurement higher in 2017 (Table 5). Moreover, the differences in bitterness were even greater (6.50 ± 0.87, 6.20 ± 0.91, and 8.49 ± 0.63 in 2015, 2016, and 2017, respectively). According to the regulation, VOO is imbalanced when the median of bitter and/or pungent is higher than the median of fruity by two or more units of measurement [63], which was the case here (Table 5). Irr 75 significantly reduced the bitterness intensity, and with the increase of water addition (Irr 100), the bitterness further decreased. The recorded results can be interpreted in two directions. First, as negative, since producers with robust and intense bitter and pungent VOOs achieve the best results in many competitions. Moreover, more intense oils/higher TCP mean that the VOOs are more stable. However, the second approach faces consumers as most of them want a balanced oil with medium intensity for daily consumption. The pungent taste and highly bitter oils are perceived negatively by consumers and represent a barrier, especially for those less familiar with VOOs [69]. Extra virgin olive oils from irrigated treatment (Irr 75, Irr 100) can be defined sensorially as balanced, harmonious oils with medium fruitiness, medium bitterness, and medium to intense pungency (Table 5).

### 3.3. Influence of Irrigation and Growing Season on Fatty Acid Profile

GC analysis of fatty acids obtained from the VOOs with different irrigation strategies showed the same profile of each fatty acid, but in different proportions (Table 6). As expected, the fatty acid profile was dominated by oleic acid (C18:1), with a mean content of 69.6%, followed by palmitic acid (12.8%) and linoleic acid with 12.53%. Data analysis (two-way ANOVA) showed that irrigation treatments significantly affected only the percentage of oleic acid and linoleic acid. Lower levels of oleic acid were found in oils from trees that received more water (Irr 75 and Irr 100). Conversely, linoleic acid content was significantly higher in oils from irrigated trees than in oils from control trees (rain-fed treatment) (Table 6). Such fatty acid changes have been observed in previous studies [5,8]. Cano-Lamadrid et al. [8] recorded an additional effect on palmitoleic fatty acid, which decreased with irrigation. A decrease in oleic fatty acid with irrigation was also observed in olive oils of Baladi and Edlbi varieties, where irrigation caused changes in six other fatty acids [70], although opposite effects of irrigation on oleic acid were also reported [65]. 

Growing season as a factor had a significant effect on the content of most of the analyzed fatty acids (the exception being gadoleic acid). The highest variability (F value) was found for palmitic acid. A significant interaction between the main factors (irrigation treatment × growing season) was observed for palmitic acid, oleic acid, and linoleic acid (Table 6). In 2016 and 2017, palmitic acid differed between treatments but without a consistent pattern of change. For the other two fatty acids mentioned, a slight, although not significant, reduction in oleic acid with irrigation was observed in 2016 (the third year of irrigation treatments). With the extension of the experiment (2017), irrigated VOOs had statistically lower levels of oleic acid (Table 6), while the opposite was true for linoleic acid. This year was also the drier year in which the fatty acid profile was most affected compared to the other two years (Figure 1). The changes visible and measurable after a few years of irrigation were also observed in the study of oils from Arbequina olive trees [71]. However, in previous work, different effects of irrigation on fatty acid composition were observed in the years studied, such as a decrease in oleic acid and an increase in palmitic and linoleic acid in one year studied and no effect or irregular changes in the second year [31,33]. In other experiments conducted over several years, the growing season proved to be a more influential factor than irrigation [10,71]. The changes in fatty acid synthesis are associated with the activity of the condensing enzyme β-ketoacyl-ACP synthase II (KAS), which catalyzes the elongation of palmitoyl-ACP to stearoyl-ACP [72]. As suggested by Beltran et al. [73], the reduced oleic acid content may be due in part to lower KAS activity in the presence of higher water availability during fruit growth and intense oil synthesis. Other environmental factors, such as light and temperature, may also influence activity [74]. 

In addition to the numbered fatty acids in Table 6, lauric acid (C 12:0, 0.02 ± 0.05), myristic acid (C 14:0, 0.02 ± 0.05), behenic acid (C 22:0, 0.10 ± 0.01), lignoceric acid (C 24:0, 0.10 ± 0.01), heptadecenoic acid (C 17:1, 0.10 ± 0.01), arachidonic acid (C 20:0, 0.40 ± 0.03), and heptadecanoic acid (C 17:0, 0.04 ± 0.01), with no differences as a function of irrigation or within growing seasons were determined. All VOOs obtained in the present study were within the regulatory limits [63]. Oblica was characterized by a medium content of oleic acid, high content of palmitic acid, and medium content of linoleic fatty acid [24]. The VOOs of Oblica cultivar obtained in the present study differed by a detected significant increase in linoleic acid (effect of growing season, and effect of irrigation), so the other listed fatty acid oils remained in the same categories, while the difference is that these oils can be called oils with high linoleic acid content.

Table 7 contains the fatty acids of the oils studied, grouped according to the degree of unsaturation. Monounsaturated and polyunsaturated fatty acids were significantly affected by the irrigation regime. Monounsaturated fatty acids were higher and polyunsaturated fatty acids lower in trees with medium and full irrigation (75% and 100% ET) than in oils from non-irrigated trees, although the increases and decreases were not linear with irrigation increase. In both groups, Irr 50 did not differ from the control treatment. The irrigation regime showed significant differences in the ratios of oleic acid/linolenic acid and monounsaturated/polyunsaturated fatty acids (MUFA/PUFA), both of which gradually decreased with increasing water supply.

The growing season affects all groups and ratios of fatty acids. MUFA and the ratio of oleic acid to linoleic acid were higher in 2017 than in 2016 or 2015, and a significant interaction between irrigation and growing season was found for PUFA, MUFA, the ratio of oleic acid to linoleic acid, and MUFA/PUFA. Irrigation treatments did not show the same response across years, with 2015 being quite erratic, while 2016 and 2017 followed the same trend. The greater decrease in the oleic/linolenic acid ratio with irrigation was observed in the year with the highest ratio (25% in 2016: 31% in 2017) (Table 7).

The ratios are positively correlated with the oxidative stability of the oil, and the average oleic acid/linolenic acid ratio determined in the present study was 5.6 (with a range of 4.3 to 8.3). A medium to low ratio is indicative of the importance of suitable storage conditions for Oblica oils, regardless of irrigation treatment (Table 7).

## 4. Conclusions

This study investigated a series of morphological characteristics, chemical properties, and sensory attributes of Oblica cultivar VOOs under the influence of four treatments: irrigation corresponding to 50%, 75%, and 100% ETc, and the control treatment as rain-fed. 

Irrigation treatments in two analyzed growing seasons influenced the measured parameters to different degrees. Thus, in conclusion, we provide descriptions of fruits and VOOs by listing the most prominent characteristics noted in each growing season and the changes observed with irrigation treatments:

- Year 2016 had 356.60 ± 66.14 fruits per kg, oil yield of 14.09 ± 1.17%, balanced VOO of medium to intense bitterness and pungency; by irrigation → increased fruit size characteristics by Irr 100 → increased fruit weight by Irr 100 → increased oil yield for 18% → irregular changes in oleic fatty acid → unchanged phenolics, bitterness and pungency.
- Year 2017 had 617.42 ± 83.69 fruits per kg, the smallest fruit proportions, the lowest oil yield (6.4 ± 0.09%), high phenolic content, unbalanced VOO with intense bitterness and pungency; due to irrigation → increased fruit size characteristics for all irrigation treatments → increased fruit weight for all irrigation treatments → increased oil yield for 35% → decreased oleic fatty acid → decreased phenols → decreased bitterness and pungency → balanced VOO achieved.

According to our results, the irrigation of olives grown on reclaimed karst soil can improve the main morphometric parameters of fruit and stone, as well as oil yield.

## Figures and Tables

**Table 1:** Experimental treatments and abbreviations used in the study.

Treatment	Abbreviation
Rain-fed as a control	C
Irrigation corresponding to 50% ETc	Irr 50
Irrigation corresponding to 75% ETc	Irr 75
Irrigation corresponding to 100% ETc	Irr 100

**Table 2:** Effect of different irrigation levels on olive fruit parameters during two experimental seasons.

Factor		Area (cm^2^)	Width (cm)	Length (cm)	Aspect Ratio (W/L)	Perimeter (cm)	Shape	Volume
2016	C	3.25 ± 0.85 b	1.95 ± 0.19 b	2.08 ± 0.35 b	0.95 ± 0.08 a	6.72 ± 0.89 b	0.9 ± 0.06 a	4.65 ± 2.19 b
	Irr 50	3.11 ± 0.62 b	1.91 ± 0.19 bc	2.06 ± 0.25 b	0.93 ± 0.07 b	6.79 ± 0.9 b	0.86 ± 0.09 b	4.36 ± 1.35 b
	Irr 75	3.07 ± 0.52 b	1.89 ± 0.17 c	2.06 ± 0.2 b	0.92 ± 0.05 b	6.89 ± 0.76 b	0.83 ± 0.13 bc	4.28 ± 1.10 b
	Irr 100	3.94 ± 0.85 a	2.11 ± 0.23 a	2.36 ± 0.27 a	0.90 ± 0.1 c	7.86 ± 1.3 a	0.82 ± 0.12 c	6.33 ± 2.12 a
2017	C	1.86 ± 0.41 d	1.49 ± 0.16 d	1.58 ± 0.2 d	0.95 ± 0.05 a	4.96 ± 0.59 d	0.95 ± 0.03 a	2.01 ± 0.71 d
	Irr 50	3.26 ± 0.72 c	1.96 ± 0.19 c	2.12 ± 0.27 c	0.93 ± 0.05 b	6.64 ± 0.75 c	0.93 ± 0.05 b	4.74 ± 1.67 c
	Irr 75	3.88 ± 0.64 b	2.09 ± 0.17 b	2.38 ± 0.24 b	0.88 ± 0.05 c	7.29 ± 0.65 b	0.92 ± 0.05 bc	6.28 ± 1.63 b
	Irr 100	4.19 ± 0.59 a	2.15 ± 0.16 a	2.50 ± 0.2 a	0.87 ± 0.05 d	7.64 ± 0.62 a	0.91 ± 0.06 c	7.13 ± 1.52 a
IRR	C	2.55 ± 0.96 d	1.72 ± 0.29 d	1.83 ± 0.38 d	0.95 ± 0.07 a	5.84 ± 1.16 d	0.92 ± 0.05 a	3.33 ± 2.1 d
	Irr 50	3.19 ± 0.67 c	1.93 ± 0.19 c	2.09 ± 0.26 c	0.93 ± 0.06 b	6.71 ± 0.83 c	0.89 ± 0.08 b	4.55 ± 1.53 c
	Irr 75	3.49 ± 0.71 b	1.99 ± 0.19 b	2.22 ± 0.27 b	0.9 ± 0.06 c	7.1 ± 0.73 b	0.87 ± 0.11 c	5.31 ± 1.72 b
	Irr 100	4.07 ± 0.74 a	2.13 ± 0.2 a	2.43 ± 0.25 a	0.88 ± 0.05 d	7.75 ± 1.02 a	0.86 ± 0.1 c	6.74 ± 1.88 a
	F	268.76	274.60	307.43	92.10	275.70	35.90	234.39
	*p*	***	***	***	***	***	***	***
GS	2016	3.34 ± 0.8	1.97 ± 0.21 a	2.14 ± 0.3	0.93 ± 0.07 a	7.06 ± 1.08 a	0.85 ± 0.11 b	4.9 ± 1.94
	2017	3.3 ± 1.08	1.92 ± 0.31 b	2.15 ± 0.42	0.91 ± 0.06 b	6.63 ± 1.22 b	0.92 ± 0.05 a	5.04 ± 2.42
	F	1.44	18.1	0.07	38.8	80.20	278.9	2.16
	*p*	ns	***	ns	***	***	***	ns
IRR × GS	F	149.62	191.1	152.44	12.1	93.11	5.2	113.53
	*p*	***	***	***	**	***	**	***

Means marked by different lowercase letters (a–c) in column (for each growing season) and for each main factor (irrigation treatment and growing season) are significantly different (Tukey’s test, *p* ≤ 0.05). Significance: ** *p* ≤ 0.01; *** *p* ≤ 0.001, ns—not significant. Identification of main factors; IRR—irrigation treatment, GS—growing season. Irrigation treatments: C—Control—rain fed treatment, Irr 50—irrigation corresponding to 50% ETc, Irr 75—irrigation corresponding to 75% ETc, Irr 100—irrigation corresponding to 100% ETc, (see also Section 2.2).

**Table 3:** Effect of different irrigation levels on olive stone parameters during two experimental seasons.

Factor		Area (cm^2^)	Width (cm)	Length (cm)	Aspect Ratio (W/L)	Perimeter (cm)	Shape	Volume
2016	C	0.74 ± 0.18 b	0.87 ± 0.1 b	1.14 ± 0.16 b	0.77 ± 0.08 ab	3.14 ± 0.42 b	0.93 ± 0.04 b	0.62 ± 0.22 b
	Irr 50	0.74 ± 0.19 b	0.87 ± 0.11 b	1.13 ± 0.16 b	0.78 ± 0.07 a	3.11 ± 0.45 b	0.95 ± 0.05 a	0.61 ± 0.23 b
	Irr 75	0.76 ± 0.18 b	0.88 ± 0.1 b	1.15 ± 0.15 b	0.77 ± 0.07 a	3.19 ± 0.42 b	0.93 ± 0.05 bc	0.63 ± 0.22 b
	Irr 100	0.88 ± 0.12 a	0.94 ± 0.07 a	1.26 ± 0.11 a	0.75 ± 0.06 b	3.47 ± 0.27 a	0.92 ± 0.04 c	0.79 ± 0.16 a
2017	C	0.55 ± 0.12 d	0.74 ± 0.07 d	1.01 ± 0.14 c	0.75 ± 0.07 a	2.9 ± 0.44 c	0.84 ± 0.13 a	0.41 ± 0.13 d
	Irr 50	0.88 ± 0.17 c	0.9 ± 0.08 c	1.34 ± 0.17 b	0.68 ± 0.07 b	3.69 ± 0.46 b	0.81 ± 0.09 a	0.87 ± 0.09 c
	Irr 75	1.04 ± 0.12 b	0.96 ± 0.06 b	1.49 ± 0.11 a	0.65 ± 0.05 c	4.3 ± 0.63 a	0.73 ± 0.13 b	1.12 ± 0.13 b
	Irr 100	1.09 ± 0.13 a	0.98 ± 0.06 a	1.53 ± 0.13 a	0.65 ± 0.05 c	4.3 ± 0.54 a	0.76 ± 0.11 b	1.21 ± 0.11 a
IRR	C	0.65 ± 0.18 d	0.81 ± 0.11 d	1.08 ± 0.17 d	0.76 ± 0.07 a	3.02 ± 0.45 d	0.89 ± 0.11 a	0.51 ± 0.11 d
	Irr 50	0.81 ± 0.19 c	0.88 ± 0.1 c	1.24 ± 0.2 c	0.73 ± 0.08 b	3.4 ± 0.54 c	0.88 ± 0.1 a	0.74 ± 0.1 c
	Irr 75	0.9 ± 0.21 b	0.92 ± 0.09 b	1.32 ± 0.22 b	0.71 ± 0.09 c	3.74 ± 0.77 b	0.83 ± 0.14 b	0.87 ± 0.14 b
	Irr 100	0.99 ± 0.17 a	0.96 ± 0.07 a	1.4 ± 0.18 a	0.7 ± 0.08 c	3.89 ± 0.6 a	0.84 ± 0.12 b	1 ± 0.12 a
	F	278.28	200.4	290.64	54.5	207.81	33.5	289.08
	*p*	***	***	***	***	***	***	***
GS	2016	0.78 ± 0.18 b	0.89 ± 0.1	1.17 ± 0.16 b	0.77 ± 0.07 a	3.23 ± 0.42 b	0.93 ± 0.05 a	0.66 ± 0.05 b
	2017	0.89 ± 0.25 a	0.9 ± 0.12	1.34 ± 0.25 a	0.68 ± 0.07 b	3.8 ± 0.78 a	0.79 ± 0.12 b	0.90 ± 0.12 a
	F	167.71	2.7	448.33	623.6	450.85	868.1	388.44
	*p*	***	ns	***	***	***	***	***
IRR × GS	F	144.27	93.4	171.02	42.3	117.93	17.8	164.93
	*p*	***	***	***	***	***	***	***

Means marked by different lowercase letters (a–c) in column (for each growing season) and for each main factor (irrigation treatment and growing season) are significantly different (Tukey’s test, *p* ≤ 0.05). Significance: *** *p* ≤ 0.001, ns—not significant. Identification of main factors; IRR—irrigation treatment, GS—growing season. Irrigation treatments: C —Control—rain fed treatment, Irr 50—irrigation corresponding to 50% ETc, Irr 75—irrigation corresponding to 75% ETc, Irr 100—irrigation corresponding to 100% ETc, (see also Section 2.2).

**Table 4:** Effect of different irrigation levels on fruit yield, number of fruits per kilogram, fruit and stone weight, pulp to pit ration and oil yield in two experimental seasons.

Factor		Fruit Yield (kg tree^−1^)	No Fruit kg^−1^	Fruit Weight (g)	Pulp Weight (g)	Stone Weight (g)	Pulp to Pit Ratio	Oil Yield %
2016	C	2.12 ± 1.43	356.60 ± 66.1	2.63 ± 0.53 b	2.41 ± 0.49 b	0.22 ± 0.09 b	10.02 ± 2.33 a	14.09 ± 1.17 b
	Irr 50	1.53 ± 0.98	369.52 ± 79.1	2.76 ± 0.52 b	2.50 ± 0.4 b	0.26 ± 0.11 b	8.68 ± 1.9 ab	14.91 ± 1.62 ab
	Irr 75	2.25 ± 1.39	373.26 ± 84.9	2.85 ± 0.54 b	2.58 ± 0.48 b	0.28 ± 0.11 b	8.17 ± 1.5 ab	17.11 ± 1.13 a
	Irr 100	2.3 ± 1.70	267.11 ± 25.8	3.79 ± 0.38 a	3.41 ± 0.38 a	0.38 ± 0.07 a	8.11 ± 0.8 b	16.97 ± 1.88 a
2017	C	0.62 ± 0.42 b	617.42 ± 83.69 a	1.38 ± 0.34 c	1.15 ± 0.31 c	0.24 ± 0.06 d	4.94 ± 1.1 b	6.40 ± 0.1 b
	Irr 50	1.86 ± 0.92 ab	362.91 ± 97.21 b	2.95 ± 0.87 b	2.54 ± 0.77 b	0.41 ± 0.11 c	6.17 ± 0.6 a	10.61 ± 1.49 a
	Irr 75	2.81 ± 2.42 a	243.7 ± 22.6 c	3.94 ± 0.66 a	3.42 ± 0.57 a	0.53 ± 0.1 b	6.59 ± 0.5 a	10.32 ± 1.94 a
	Irr 100	0.84 ± 0.72 b	229.64 ± 28.99 c	4.41 ± 0.53 a	3.78 ± 0.48 a	0.64 ± 0.06 a	5.98 ± 0.4 a	9.48 ± 0.2 a
IRR	C	1.37 ± 1.29 b	431.12 ± 142.41 a	1.92 ± 0.76 d	1.69 ± 0.75 d	0.23 ± 0.07 d	7.12 ± 3.0	10.25 ± 4.13 b
	Irr 50	1.69 ± 0.95 ab	366.21 ± 83.66 ab	2.85 ± 0.71 c	2.52 ± 0.62 c	0.34 ± 0.13 c	7.42 ± 1.9	12.76 ± 2.7 a
	Irr 75	2.53 ± 1.96 a	308.48 ± 89.98 bc	3.40 ± 0.8 b	3.00 ± 0.67 b	0.40 ± 0.1 b	7.38 ± 1.3	13.72 ± 3.88 a
	Irr 100	1.57 ± 1.48 b	248.37 ± 32.55 c	4.10 ± 0.5 a	3.60 ± 0.4 a	0.51 ± 0.15 a	7.04 ± 1.2	13.64 ± 4.17 a
	F	4.1365	17.4060	60.078	56.443	45.380	0.670	12.413
	*p*	***	***	***	***	***	ns	***
GS	2016	2.05 ± 1.40 a	341.62 ± 76.73	3.05 ± 0.67	2.76 ± 0.6	0.29 ± 0.11 b	8.6 ± 1.7 a	15.77 ± 1.91 a
	2017	1.53 ± 1.59 b	318.59 ± 138.35	3.27 ± 1.29	2.81 ± 1.13	0.47 ± 0.17 a	5.97 ± 0.9 b	9.19 ± 2.1 b
	F	4.1825	0.9335	2.358	0.000	101.844	133.863	219.620
	*p*	**	ns	ns	ns	***	***	***
IRR × GS	F	4.9211	11.0714	18.338	18.384	9.332	8.545	3.152
	*p*	***	***	***	***	***	***	**

Means marked by different lowercase letters (a–c) in column (for each growing season) and for each main factor (irrigation treatment and growing season) are significantly different (Tukey’s test, *p* ≤ 0.05). Significance: ** *p* ≤ 0.01; *** *p* ≤ 0.001, ns—not significant. Identification of main factors; IRR—irrigation treatment, GS—growing season. Irrigation treatments: C—Control—rain fed treatment, Irr 50—irrigation corresponding to 50% ETc, Irr 75—irrigation corresponding to 75% ETc, Irr 100—irrigation corresponding to 100% ETc, (see also Section 2.2).

**Table 5:** Effect of different irrigation levels on quality paremetrs, phenols and sensory properties in virgin olive oils in three experimental seasons.

Factor		FFA (%)	PV (meq O_2_ kg^−1^)	K_232_	K_270_	Phenols (mg kg^−1^)	Fruitiness	Bitterness	Pungency
2015	C	0.50 ± 0.01 a	4.06 ± 0.16 bc	2.40 ± 0.01 a	0.22 ± 0.01 a	641.9 ± 10.2 a	6.17 ± 0.3 a	5.91 ± 0.8	6.47 ± 0.5
	Irr 50	0.46 ± 0.01 b	3.89 ± 0.19 c	2.34 ± 0.05 a	0.22 ± 0.01 a	610.1 ± 12.5 b	4.50 ± 0.2 c	6.84 ± 0.3	6.50 ± 0.5
	Irr 75	0.47 ± 0.01 b	4.53 ± 0.29 b	2.22 ± 0.01 b	0.19 ± 0.01 b	515.5 ± 12.8 d	5.67 ± 0.3 ab	6.00 ± 0.5	5.67 ± 0.3
	Irr 100	0.43 ± 0.02 c	5.26 ± 0.09 a	2.35 ± 0.01 a	0.21 ± 0.01 a	580.9 ± 7.5 c	4.84 ± 0.8 bc	5.50 ± 0.5	5.67 ± 0.6
2016	C	0.30 ± 0.07	5.79 ± 1.72	2.17 ± 0.21	0.20 ± 0.02	569.2 ± 148.4	4.94 ± 0.3	6.10 ± 0.9	6.54 ± 0.3
	Irr 50	0.33 ± 0.05	6.17 ± 2.01	2.14 ± 0.24	0.18 ± 0.03	578.0 ± 122.4	4.72 ± 0.8	6.04 ± 1.5	6.62 ± 0.8
	Irr 75	0.34 ± 0.05	6.16 ± 1.76	2.16 ± 0.15	0.20 ± 0.03	578.20 ± 113.2	4.56 ± 0.9	6.28 ± 0.9	6.58 ± 0.9
	Irr 100	0.35 ± 0.06	6.24 ± 1.39	2.06 ± 0.16	0.19 ± 0.03	570.8 ± 74.8	4.78 ± 0.7	6.68 ± 1.7	6.92 ± 0.5
2017	C	0.34 ± 0.02 a	6.75 ± 0.5 a	2.59 ± 0.07 a	0.29 ± 0.03 a	1099.9 ± 152.2 a	5.45 ± 0.6 a	8.49 ± 0.6 a	7.4 ± 0.4 a
	Irr 50	0.31 ± 0.04 a	3.86 ± 1.14 b	2.38 ± 0.10 b	0.22 ± 0.04 b	940.4 ± 124.9 ab	4.70 ± 0.5 a	8.15 ± 0.6 a	7.7 ± 0.6 a
	Irr 75	0.31 ± 0.04 a	5.48 ± 1.49 ab	2.20 ± 0.16 c	0.20 ± 0.04 b	801.3 ± 109.8 b	4.90 ± 0.7 a	6.02 ± 0.5 b	6.58 ± 0.8 ab
	Irr 100	0.23 ± 0.02 b	6.86 ± 2.37 a	2.35 ± 0.16 bc	0.21 ± 0.02 b	393.7 ± 114.9 c	4.33 ± 0.9 b	4.07 ± 0.5 c	5.57 ± 0.7 b
IRR	C	0.36 ± 0.09 a	5.99 ± 1.38 a	2.43 ± 0.22 a	0.25 ± 0.05 a	779.5 ± 276.1 a	5.45 ± 0.6 a	6.81 ± 1.3 a	6.76 ± 0.5 ab
	Irr 50	0.35 ± 0.07 ab	4.59 ± 1.70 b	2.30 ± 0.18 b	0.21 ± 0.04 b	731.5 ± 203.0 ab	4.70 ± 0.5 b	7.04 ± 1.4 a	7.01 ± 0.8 a
	Irr 75	0.35 ± 0.07 ab	5.51 ± 1.49 ab	2.19 ± 0.14 b	0.20 ± 0.03 b	675.8 ± 160.9 b	4.84 ± 0.8 ab	6.13 ± 0.7 ab	6.35 ± 0.8 ab
	Irr 100	0.32 ± 0.09 b	6.3 ± 1.78 a	2.25 ± 0.20 b	0.20 ± 0.03 b	502.6 ± 121.7 c	4.33 ± 0.9 b	5.52 ± 1.6 b	6.16 ± 0.9 b
	F	3.944	2.459	4.740	7.111	15.630	9.622	7.684	5.408
	*p*	**	ns	***	***	***	***	***	**
GS	2015	0.47 ± 0.03 a	4.44 ± 0.58 b	2.33 ± 0.08 a	0.21 ± 0.01 b	587.1 ± 49.1 b	5.3 ± 0.8 a	6.18 ± 0.7	6.08 ± 0.5 b
	2016	0.33 ± 0.06 b	6.09 ± 1.60 a	2.13 ± 0.18 b	0.19 ± 0.03 b	574.1 ± 108.1 b	4.75 ± 0.6 b	6.03 ± 1.2	6.67 ± 0.6 a
	2017	0.31 ± 0.05 b	5.69 ± 1.82 a	2.38 ± 0.19 a	0.23 ± 0.05 a	796.7 ± 275.08 a	4.58 ± 0.8 b	6.66 ± 1.9	6.83 ± 1.1 a
	F	98.235	5.3684	20.790	19.514	34.686	5.310	0.991	5.217
	*p*	***	***	***	***	***	***	ns	**
IRR × GS	F	5.768	1.9371	2.290	5.097	13.695	4.044	6.416	3.687
	*p*	***	ns	*	***	***	***	***	**

Means marked by different lowercase letters (a–c) in column (for each growing season) and for each main factor (irrigation treatment and growing season) are significantly different (Tukey’s test, *p* ≤ 0.05). Significance: * *p* ≤ 0.05; ** *p* ≤ 0.01; *** *p* ≤ 0.001, ns—not significant. Identification of main factors; IRR—irrigation treatment, GS—growing season. Irrigation treatments: C—Control—rain fed treatment, Irr 50—irrigation corresponding to 50% ETc, Irr 75—irrigation corresponding to 75% ETc, Irr 100—irrigation corresponding to 100% ETc, (see also Section 2.2). FFA—free fatty acids, PV—peroxide value, K_232_ and K_270_—spectrophotometric indices. Delta—K values for all analyzed samples were ≤0.01.

**Table 6:** Effect of different irrigation levels on fatty acid composition in virgin olive oils in three experimental seasons.

Factor		Palmitic (C 16:0)	Palmitoleic (C 16:1)	Stearic (C 18:0)	Oleic (C 18:1)	Linoleic (C 18:2)	Linolenic (C 18:3)	Gadoleic (C 20:1)
2015	C	14.86 ± 0.37	1.13 ± 0.26	2.29 ± 0.04	67.37 ± 0.21 b	12.44 ± 0.07 b	0.81 ± 0.01	0.30 ± 0.01
	Irr 50	14.45 ± 0.35	1.01 ± 0.22	2.3 ± 0.05	68.53 ± 0.23 a	11.82 ± 0.09 c	0.81 ± 0.01	0.31 ± 0.01
	Irr 75	14.41 ± 0.39	1.01 ± 0.23	2.23 ± 0.05	66.60 ± 0.21 c	13.82 ± 0.08 a	0.81 ± 0.01	0.31 ± 0.01
	Irr 100	14.27 ± 0.38	1.02 ± 0.23	2.22 ± 0.05	68.46 ± 0.21 a	12.30 ± 0.06 b	0.81 ± 0.01	0.31 ± 0.01
2016	C	11.56 ± 0.55 b	0.74 ± 0.09	2.64 ± 0.34	71.54 ± 1.85 a	11.80 ± 0.81 b	0.64 ± 0.09	0.32 ± 0.05
	Irr 50	11.76 ± 0.38 ab	0.70 ± 0.08	2.30 ± 0.08	70.26 ± 1.06 ab	13.32 ± 0.78 ab	0.66 ± 0.06	0.32 ± 0.05
	Irr 75	12.24 ± 0.24 a	0.72 ± 0.05	2.52 ± 0.32	68.64 ± 1.35 b	14.24 ± 1.00 a	0.66 ± 0.06	0.34 ± 0.06
	Irr 100	12.10 ± 0.20 ab	0.70 ± 0.08	2.26 ± 0.22	69.38 ± 1.16 ab	13.84 ± 1.20 a	0.66 ± 0.06	0.32 ± 0.05
2017	C	12.25 ± 0.22	1.00 ± 0.00 a	2.10 ± 0.00 b	73.80 ± 0.99 a	8.90 ± 0.71 b	0.80 ± 0.00 a	0.30 ± 0.00
	Irr 50	13.00 ± 0.25	0.94 ± 0.12 a	2.22 ± 0.05 ab	70.64 ± 0.46 b	11.44 ± 0.49 a	0.68 ± 0.05 b	0.30 ± 0.00
	Irr 75	12.76 ± 0.27	0.80 ± 0.00 b	2.26 ± 0.06 a	70.22 ± 0.71 b	12.16 ± 0.58 a	0.70 ± 0.00 b	0.34 ± 0.06
	Irr 100	12.37 ± 0.47	0.77 ± 0.06 b	2.30 ± 0.10 a	70.64 ± 0.87 b	12.07 ± 0.52 a	0.70 ± 0.00 b	0.34 ± 0.06
IRR	C	12.69 ± 1.58	0.91 ± 0.23	2.43 ± 0.33	70.74 ± 2.81 a	11.42 ± 1.48 c	0.73 ± 0.11	0.31 ± 0.04
	Irr 50	12.86 ± 1.11	0.87 ± 0.19	2.27 ± 0.07	70.01 ± 1.09 ab	12.25 ± 1.04 b	0.71 ± 0.08	0.31 ± 0.03
	Irr 75	12.94 ± 0.91	0.82 ± 0.15	2.36 ± 0.24	68.78 ± 1.69 c	13.35 ± 1.19 a	0.71 ± 0.07	0.34 ± 0.05
	Irr 100	12.77 ± 1.02	0.81 ± 0.18	2.26 ± 0.16	69.48 ± 1.19 bc	12.94 ± 1.18 ab	0.72 ± 0.08	0.32 ± 0.05
	F	1.280	1.983	0.607	9.910	19.410	0.950	0.770
	*p*	ns	ns	ns	***	***	ns	ns
GS	2015	14.50 ± 0.39 a	1.05 ± 0.21 a	2.26 ± 0.06 b	67.74 ± 0.87 c	12.60 ± 0.78 b	0.81 ± 0.01 a	0.31 ± 0.01
	2016	11.92 ± 0.44 c	0.72 ± 0.07 c	2.43 ± 0.29 a	69.96 ± 1.69 b	13.30 ± 1.30 a	0.66 ± 0.07 c	0.33 ± 0.05
	2017	12.70 ± 0.40 b	0.87 ± 0.12 b	2.24 ± 0.09 b	70.92 ± 1.34 a	11.47 ± 1.20 c	0.71 ± 0.05 b	0.32 ± 0.05
	F	211.231	25.281	6.618	39.301	35.761	38.220	1.391
	*p*	***	***	***	***	***	***	ns
IRR × GS	F	3.730	0.765	2.014	3.211	3.390	1.580	0.271
	*p*	**	ns	ns	*	**	ns	ns

Means marked by different lowercase letters (a–c) in column (for each growing season) and for each main factor (irrigation treatment and growing season) are significantly different (Tukey’s test, *p* ≤ 0.05). Significance: * *p* ≤ 0.05; ** *p* ≤ 0.01; *** *p* ≤ 0.001, ns—not significant. Identification of main factors; IRR—irrigation treatment, GS—growing season. Irrigation treatments: C—Control—rain fed treatment, Irr 50—irrigation corresponding to 50% ETc, Irr 75—irrigation corresponding to 75% Etc, Irr 100—irrigation corresponding to 100% Etc, (see also Section 2.2).

**Table 7:** Effect of different irrigation levels on fatty acids grouped upon unsaturation degree and their ratios in virgin olive oils in three experimental seasons.

Factor		ΣSFA	ΣPUFA	ΣMUFA	Oleic/Linoleic Ratio	MUFA/SFA	MUFA/PUFA
2015	C	17.75 ± 0.43	13.25 ± 0.08 b	68.90 ± 0.47 ab	5.42 ± 0.02 c	3.89 ± 0.07	5.21 ± 0.02 c
	Irr 50	17.34 ± 0.28	12.62 ± 0.09 c	69.95 ± 0.46 a	5.81 ± 0.03 a	4.04 ± 0.09	5.55 ± 0.01 a
	Irr 75	17.23 ± 0.32	14.62 ± 0.07 a	68.01 ± 0.44 b	4.83 ± 0.02 d	3.95 ± 0.10	4.66 ± 0.02 d
	Irr 100	17.08 ± 0.32	13.11 ± 0.07 b	69.88 ± 0.43 a	5.57 ± 0.02 b	4.10 ± 0.10	5.34 ± 0.01 b
2016	C	14.82 ± 0.94	12.44 ± 0.79 b	72.70 ± 1.80 a	6.10 ± 0.56 a	4.93 ± 0.42	5.87 ± 0.51
	Irr 50	14.68 ± 0.41	13.98 ± 0.74 ab	71.38 ± 1.11 ab	5.30 ± 0.40 ab	4.87 ± 0.21	5.13 ± 0.36
	Irr 75	15.38 ± 0.55	14.90 ± 1.04 a	69.80 ± 1.33 b	4.85 ± 0.43 b	4.55 ± 0.23	4.71 ± 0.41
	Irr 100	14.98 ± 0.39	14.50 ± 1.26 a	70.50 ± 1.11 ab	5.05 ± 0.54 b	4.71 ± 0.14	4.90 ± 0.52
2017	C	15.05 ± 0.22 b	9.70 ± 0.71 b	75.20 ± 0.99 a	8.33 ± 0.78 a	5.00 ± 0.14 a	7.78 ± 0.67 a
	Irr 50	15.84 ± 0.28 a	12.12 ± 0.47 a	71.98 ± 0.47 b	6.19 ± 0.29 b	4.55 ± 0.09 b	5.95 ± 0.26 b
	Irr 75	15.64 ± 0.33 ab	12.86 ± 0.58 a	71.46 ± 0.71 b	5.79 ± 0.35 b	4.58 ± 0.13 b	5.57 ± 0.32 b
	Irr 100	15.27 ± 0.38 ab	12.77 ± 0.52 a	71.84 ± 0.77 b	5.87 ± 0.33 b	4.71 ± 0.17 ab	5.64 ± 0.29 b
IRR	C	15.75 ± 1.54	12.14 ± 1.46 c	72.06 ± 2.72 a	6.34 ± 1.19 a	4.63 ± 0.59	6.06 ± 1.05 a
	Irr 50	15.74 ± 1.10	12.95 ± 1.01 bc	71.28 ± 1.09 ab	5.76 ± 0.50 b	4.56 ± 0.36	5.54 ± 0.46 b
	Irr 75	15.91 ± 0.86	14.06 ± 1.20 a	70.03 ± 1.64 c	5.21 ± 0.58 c	4.42 ± 0.31	5.03 ± 0.54 c
	Irr 100	15.63 ± 1.00	13.65 ± 1.17	ab 70.70 ± 1.12	bc 5.42 ± 0.52	bc 4.55 ± 0.32	5.22 ± 0.48 bc
	F	0.850	18.631	10.600	25.014	2.780	23.670
	*p*	ns	***	***	***	ns	***
GS	2015	17.35 ± 0.39 a	13.40 ± 0.78 a	69.19 ± 0.92 c	5.41 ± 0.38 b	4.00 ± 0.12 b	5.19 ± 0.35 b
	2016	14.97 ± 0.63 c	13.96 ± 1.31 a	71.10 ± 1.68 b	5.32 ± 0.66 b	4.77 ± 0.29 a	5.15 ± 0.62 b
	2017	15.56 ± 0.40 b	12.18 ± 1.17 b	72.21 ± 1.38 a	6.28 ± 0.92 a	4.65 ± 0.19 a	6.01 ± 0.80 a
	F	97.630	33.360	35.600	43.092	63.140	41.020
	*p*	***	***	***	***	***	***
IRR × GS	F	1.990	3.180	3.100	6.464	2.140	5.720
	*p*	ns	*	*	***	ns	***

Means marked by different lowercase letters (a–c) in column (for each growing season) and for each main factor (irrigation treatment and growing season) are significantly different (Tukey’s test, *p* ≤ 0.05). Significance: * *p* ≤ 0.05; *** *p* ≤ 0.001, ns—not significant. Identification of main factors; IRR—irrigation treatment, GS—growing season. Irrigation treatments: C—Control—rain fed treatment, Irr 50—irrigation corresponding to 50% ETc, Irr 75—irrigation corresponding to 75% ETc, Irr 100—irrigation corresponding to 100% ETc, (see also Section 2.2). ΣSFA—saturated fatty acid; ΣPUFA—polyunsaturated fatty acid; ΣMUFA—monounsaturated fatty acid.

## Data Availability

The original contributions generated for this study are included in the article/Supplementary Material; further inquiries can be directed to the corresponding author.

## Supplementary Materials

The following supporting information can be downloaded at: www.mdpi.com/article/10.3390/foods11182923/s1, Figure S1: Monthly mean temperature, precipitation sum and evapotranspiration during the (a) 2015, (b) 2016, (c) 2017 and (d) Walter-Lieth climate diagram.

## References

[1] Sojka R.E., Bjorneberg D.L., Entry J.A. (2017). Irrigation: Historical Perspective. Encyclopedia of Soil Science.

[2] López-Gunn E., Mayor B., Dumont A. (2012). Implications of the Modernization of Irrigation Systems. Water, Agriculture and the Environment in Spain: Can We Square the Circle?.

[3] Connor D.J., Fereres E. (2010). The Physiology of Adaptation and Yield Expression in Olive. Hortic. Rev. (Am. Soc. Hortic. Sci.).

[4] Fraga H., Moriondo M., Leolini L., Santos J.A. (2021). Mediterranean Olive Orchards under Climate Change: A Review of Future Impacts and Adaptation Strategies. Agronomy.

[5] Fregapane G., Gómez-Rico A., Salvador M.D. (2010). Influence of Irrigation Management and Ripening on Virgin Olive Oil Quality and Composition. Olives and Olive Oil in Health and Disease Prevention.

[6] Blazakis K.N., Kosma M., Kostelenos G., Baldoni L., Bufacchi M., Kalaitzis P. (2017). Description of olive morphological parameters by using open access software. Plant Methods.

[7] Zhao C., Zhang Y., Du J., Guo X., Wen W., Gu S., Wang J., Fan J. (2019). Crop Phenomics: Current Status and Perspectives. Front. Plant Sci..

[8] Cano-Lamadrid M., Girón I.F., Pleite R., Burló F., Corell M., Moriana A., Carbonell-Barrachina A. (2015). Quality attributes of table olives as affected by regulated deficit irrigation. LWT Food Sci. Technol..

[9] D’Andria R., Lavini A., Morelli G., Sebastiani L., Tognetti R. (2009). Physiological and productive responses of *Olea europaea* L. cultivars Frantoio and Leccino to a regulated deficit irrigation regime. Plant Biosyst..

[10] Caruso G., Gucci R., Urbani S., Esposto S., Taticchi A., Di Maio I., Selvaggini R., Servili M. (2014). Effect of different irrigation volumes during fruit development on quality of virgin olive oil of cv. Frantoio. Agric. Water Manag..

[11] Gucci R., Lodolini E., Rapoport H.F. (2007). Productivity of olive trees with different water status and crop load. J. Hortic. Sci. Biotechnol..

[12] Gomez del Campo M. (2005). Summer Deficit-Irrigation Strategies in a Hedgerow Olive Orchard Cv. ‘Arbequina’: Effect on Fruit Characteristics and Yield. Irrig. Sci..

[13] Rondanini D.P., Castro D.N., Searles P.S., Rousseaux M.C. (2014). Contrasting patterns of fatty acid composition and oil accumulation during fruit growth in several olive varieties and locations in a non-Mediterranean region. Eur. J. Agron..

[14] Faghim J., Mohamed M.B., Bagues M., Guasmi F., Triki T., Nagaz K. (2021). Irrigation effects on phenolic profile and extra virgin olive oil quality of “Chemlali” variety grown in South Tunisia. South Afr. J. Bot..

[15] Machado M., Felizardo C., Fernandes-Silva A.A., Nunes F.M., Barros A. (2013). Polyphenolic compounds, antioxidant activity and l-phenylalanine ammonia-lyase activity during ripening of olive cv. “Cobrançosa” under different irrigation regimes. Food Res. Int..

[16] Dabbou S., Rjiba I., Nakbi A., Gazzah N., Issaoui M., Hammami M. (2010). Compositional quality of virgin olive oils from cultivars introduced in Tunisian arid zones in comparison to Chemlali cultivars. Sci. Hortic..

[17] Spika M.J., Liber Z., Montemurro C., Miazzi M.M., Ljubenkov I., Soldo B., Žanetić M., Vitanović E., Politeo O., Škevin D. (2022). Quantitatively Unraveling Hierarchy of Factors Impacting Virgin Olive Oil Phenolic Profile and Oxidative Stability. Antioxidants.

[18] Romić D., Kontić J.K., Preiner D., Romić M., Lazarević B., Maletić E., Ondrašek G., Andabaka Ž., Begić H.B., Kovačić M.B. (2020). Performance of grapevine grown on reclaimed Mediterranean karst land: Appearance and duration of high temperature events and effects of irrigation. Agric. Water Manag..

[19] Kottek M., Grieser J., Beck C., Rudolf B., Rubel F. (2006). World map of the Köppen-Geiger climate classification updated. Meteorol. Z..

[20] Walter H., Lieth H. (1967). Klima-Diagramm Weltatlas.

[21] Allen R.G., Pereira L.S., Raes D., Smith M., Ab W. (1998). Guidelines for Computing Crop Water Requirements—FAO. FAO Irrig. Drain. Pap..

[22] Fernández J.E., Diaz-Espejo A., Infante J.M., Durán P., Palomo M.J., Chamorro V., Girón I., Villagarcía L. (2006). Water relations and gas exchange in olive trees under regulated deficit irrigation and partial rootzone drying. Plant Soil.

[23] Uceda M., Frias L. Harvest Dates. Evolution of the Fruit Oil Content, Oil Composition and Oil Quality. Proceedings of the Del Segundo Seminario Oleicola Internacional—COI.

[24] Špika M.J., Perica S., Žanetić M., Škevin D. (2021). Virgin Olive Oil Phenols, Fatty Acid Composition and Sensory Profile: Can Cultivar Overpower Environmental and Ripening Effect?. Antioxidants.

[25] EEC (1991). Characteristics of Olive Oil and Olive-Residue Oil and the Relevant Methods of Analysis. Regulation EEC/2568/91 and Later Modifications. Off. J. Eur. Community.

[26] (2007). Organoleptic Assessment of Virgin Olive Oil.

[27] Gutfinger T. (1981). Polyphenols in olive oils. J. Am. Oil Chem. Soc..

[28] (1990). Animal and Vegetable Fats and Oils—Analysis by Gas Chromatography of Methyl Esters of Fatty Acids.

[29] (2011). Animal and Vegetable Fats and Oils—Gas Chromatography of Fatty Acid Methyl Esters—Part 2: Preparation of Methyl Esters of Fatty Acids.

[30] Marcelić Š., Vidovi N., Pasković I., Lukić M., Špika M.J., Palčić I., Lukić I., Petek M., Pecina M., Ćustić M.H. (2022). Combined Sulfur and Nitrogen Foliar Application Increases Extra Virgin Olive Oil Quantity without Affecting Its Nutritional Quality. Horticulturae.

[31] Morales-Sillero A., García J.M., Torres-Ruiz J.M., Montero A., Sánchez-Ortiz A., Fernández J.E. (2013). Is the productive performance of olive trees under localized irrigation affected by leaving some roots in drying soil?. Agric. Water Manag..

[32] Moriana A., Orgaz F., Pastor M., Fereres E. (2003). Yield Responses of a Mature Olive Orchard to Water Deficits. J. Am. Soc. Hortic. Sci..

[33] Gómez-Rico A., Salvador M.D., Moriana A., Perez-Lopez D., Olmedilla N., Ribas F., Fregapane G. (2007). Influence of different irrigation strategies in a traditional Cornicabra cv. olive orchard on virgin olive oil composition and quality. Food Chem..

[34] Lavee S., Hanoch E., Wodner M., Abramowitch H. (2007). The effect of predetermined deficit irrigation on the performance of cv. Muhasan olives (*Olea europaea* L.) in the eastern coastal plain of Israel. Sci. Hortic..

[35] Caruso G., Rapoport H.F., Gucci R. (2013). Long-term evaluation of yield components of young olive trees during the onset of fruit production under different irrigation regimes. Irrig. Sci..

[36] Iniesta F., Testi L., Orgaz F., Villalobos F. (2009). The effects of regulated and continuous deficit irrigation on the water use, growth and yield of olive trees. Eur. J. Agron..

[37] Perez-Lopez D., Ribas F., Moriana A., Olmedilla N., de Juan A. (2007). The effect of irrigation schedules on the water relations and growth of a young olive (*Olea europaea* L.) orchard. Agric. Water Manag..

[38] Servili M., Esposto S., Lodolini E., Selvaggini R., Taticchi A., Urbani S., Montedoro G., Serravalle M., Gucci R. (2007). Irrigation Effects on Quality, Phenolic Composition, and Selected Volatiles of Virgin Olive Oils Cv. Leccino. J. Agric. Food Chem..

[39] Dabbou S., Chehab H., Faten B., Dabbou S., Esposto S., Selvaggini R., Taticchi A., Servili M., Montedoro G.F., Hammami M. (2010). Effect of three irrigation regimes on Arbequina olive oil produced under Tunisian growing conditions. Agric. Water Manag..

[40] Morales-Sillero A., Jiménez R., Fernández J.E., Troncoso A., Beltrán G. (2007). Influence of Fertigation in ‘Manzanilla de Sevilla’ Olive Oil Quality. HortScience.

[41] Patumi M., D’Andria R., Marsilio V., Fontanazza G., Morelli G., Lanza B. (2002). Olive and olive oil quality after intensive monocone olive growing (*Olea europaea* L., cv. Kalamata) in different irrigation regimes. Food Chem..

[42] García J.M., Cuevas M.V., Fernández J.E. (2013). Production and oil quality in ‘Arbequina’ olive (*Olea europaea* L.) trees under two deficit irrigation strategies. Irrig. Sci..

[43] Špika M.J., Žanetić M., Kraljic K., Pasković I., Škevin D. (2018). Changes in olive fruit characteristics and oil accumulation in ‘Oblica’ and ‘Leccino’ during ripening. Acta Hortic..

[44] Ramos A.F., Santos F.L. (2010). Yield and olive oil characteristics of a low-density orchard (cv. Cordovil) subjected to different irrigation regimes. Agric. Water Manag..

[45] Dag A., Kerem Z., Yogev N., Zipori I., Lavee S., Ben-David E. (2011). Influence of time of harvest and maturity index on olive oil yield and quality. Sci. Hortic..

[46] Berenguer M.J., Vossen P.M., Grattan S.R., Connell J.H., Polito V.S. (2006). Tree Irrigation Levels for Optimum Chemical and Sensory Properties of Olive Oil. HortScience.

[47] Ben-Gal A., Yermiyahu U., Zipori I., Presnov E., Hanoch E., Dag A. (2011). The influence of bearing cycles on olive oil production response to irrigation. Irrig. Sci..

[48] Baccouri O., Guerfel M., Bonoli-Carbognin M., Cerretani L., Bendini A., Zarrouk M., Daoud D. (2009). Influence of Irrigation and Site of Cultivation on Qualitative and Sensory Characteristics of a Tunisian Minor Olive Variety (Cv. Marsaline). Riv. Ital. Sostaze Grasse.

[49] Inglese P., Barone E., Gullo G. (1996). The Effect of Complementary Irrigation on Fruit Growth, Ripening Pattern and Oil Characteristics of Olive (*Olea Europaea* L.) Cv. Carolea. J. Hortic. Sci. Biotechnol..

[50] Gucci R., Caruso G., Gennai C., Esposto S., Urbani S., Servili M. (2019). Fruit growth, yield and oil quality changes induced by deficit irrigation at different stages of olive fruit development. Agric. Water Manag..

[51] Jesus Tovar M., Paz Romero M., Girona J., Jos Motilva M. (2002). L-Phenylalanine ammonia-lyase activity and concentration of phenolics in developing olive (*Olea europaea* L cv Arbequina) fruit grown under different irrigation regimes. J. Sci. Food Agric..

[52] García-Rodríguez R., Romero-Segura C., Sanz C., Sánchez-Ortiz A., Pérez A.G. (2011). Role of polyphenol oxidase and peroxidase in shaping the phenolic profile of virgin olive oil. Food Res. Int..

[53] Romero-Segura C., García-Rodríguez R., Sánchez-Ortiz A., Sanz C., Pérez A.G. (2012). The role of olive β-glucosidase in shaping the phenolic profile of virgin olive oil. Food Res. Int..

[54] Ilyasoğlu H., Ozcelik B., Van Hoed V., Verhé R. (2010). Characterization of Aegean Olive Oils by Their Minor Compounds. J. Am. Oil Chem. Soc..

[55] Bedbabis S., Ferrara G. (2018). Effects of long-term irrigation with treated wastewater on leaf mineral element contents and oil quality in Olive cv. Chemlali. J. Hortic. Sci. Biotechnol..

[56] El Riachy M., Priego-Capote F., León L., Rallo L., Luque de Castro M.D. (2011). Hydrophilic antioxidants of virgin olive oil. Part 2: Biosynthesis and biotransformation of phenolic compounds in virgin olive oil as affected by agronomic and processing factors. Eur. J. Lipid Sci. Technol..

[57] Diamantakos P., Ioannidis K., Papanikolaou C., Tsolakou A., Rigakou A., Melliou E., Magiatis P. (2021). A New Definition of the Term “High-Phenolic Olive Oil” Based on Large Scale Statistical Data of Greek Olive Oils Analyzed by qNMR. Molecules.

[58] Panel E., Nda A. (2011). Scientific Opinion on the Substantiation of Health Claims Related to Polyphenols in Olive and Protection of LDL Particles from Oxidative Damage (ID 1333, 1638, 1639, 1696, 2865), Maintenance of Normal Blood HDL Cholesterol Concentrations (ID 1639), Mainte. EFSA J..

[59] Martín-Peláez S., Covas M.I., Fitó M., Kušar A., Pravst I. (2013). Health effects of olive oil polyphenols: Recent advances and possibilities for the use of health claims. Mol. Nutr. Food Res..

[60] Bendini A., Cerretani L., Carrasco-Pancorbo A., Gómez-Caravaca A.M., Segura-Carretero A., Fernández-Gutiérrez A., Lercker G., Simal-Gandara J. (2007). Phenolic Molecules in Virgin Olive Oils: A Survey of Their Sensory Properties, Health Effects, Antioxidant Activity and Analytical Methods. An Overview of the Last Decade. Molecules.

[61] Angerosa F., Mostallino R., Basti C., Vito R. (2000). Virgin olive oil odour notes: Their relationships with volatile compounds from the lipoxygenase pathway and secoiridoid compounds. Food Chem..

[62] Cecchi L., Migliorini M., Mulinacci N. (2021). Virgin Olive Oil Volatile Compounds: Composition, Sensory Characteristics, Analytical Approaches, Quality Control, and Authentication. J. Agric. Food Chem..

[63] European Union Commission (2016). Commission Delegated Regulation No. 2016/2095 of 26 September 2016 Amending Regulation (EEC) No. 2568/91 on the Characteristics of Olive Oil and Olive-Residue Oil and on the Relevant Methods of Analysis. Off. J. Eur. Union.

[64] Žanetić M., Špika M.J., Ožić M.M., Bubola K.B. (2021). Comparative Study of Volatile Compounds and Sensory Characteristics of Dalmatian Monovarietal Virgin Olive Oils. Plants.

[65] Bubola K.B., Kolega Š., Marcelić Š., Šikić G., Pinto A.G., Zorica M., Klisović D., Novoselić A., Špika M.J., Kos T. (2022). Effect of Different Watering Regimes on Olive Oil Quality and Composition of Coratina Cultivar Olives Grown on Karst Soil in Croatia. Foods.

[66] Tovar M.J., Motilva M.J., Romero M.P. (2001). Changes in the Phenolic Composition of Virgin Olive Oil from Young Trees (*Olea europaea* L. cv. Arbequina) Grown under Linear Irrigation Strategies. J. Agric. Food Chem..

[67] Dabbou S., Brahmi F., Selvaggini R., Chehab H., Taticchi A., Servili M., Hammami M. (2011). Contribution of irrigation and cultivars to volatile profile and sensory attributes of selected virgin olive oils produced in Tunisia. Int. J. Food Sci. Technol..

[68] Fernandes-Silva A.A., Falco V., Correia C., Villalobos F. (2013). Sensory analysis and volatile compounds of olive oil (cv. Cobrançosa) from different irrigation regimes. Grasas Aceites.

[69] Recchia A., Monteleone E., Tuorila H. (2012). Responses to extra virgin olive oils in consumers with varying commitment to oils. Food Qual. Prefer..

[70] El Riachy M., Haber A., Daya S.A., Jebbawi G., Al Hawi G., Talej V., Houssein M., El Hajj A. (2017). Influence of Irrigation Regimes on Quality Attributes of Olive Oils from Two Varieties Growing in Lebanon. Int. J. Environ. Agric. Biotechnol..

[71] Tovar M.J., Romero-Fabregat M.-P., Alegre S.M., Girona J., Motilva M.J. (2002). Composition and organoleptic characteristics of oil fromArbequina olive (*Olea europaea* L.) trees under deficit irrigation. J. Sci. Food Agric..

[72] Salas J.J., Sánchez J., Ramli U.S., Manaf A.M., Williams M., Harwood J.L. (2000). Biochemistry of lipid metabolism in olive and other oil fruits. Prog. Lipid Res..

[73] Beltrán G., del Rio C., Sánchez S., Martínez L. (2004). Influence of Harvest Date and Crop Yield on the Fatty Acid Composition of Virgin Olive Oils from Cv. Picual. J. Agric. Food Chem..

[74] Sánchez J., Harwood J.L. (2002). Biosynthesis of triacylglycerols and volatiles in olives. Eur. J. Lipid Sci. Technol..

