# Supplementary material for: Irrigation of Young Olives Grown on Reclaimed Karst Soil Increases Fruit Size, Weight and Oil Yield and Balances the Sensory Oil Profile
Source: Foods. 2022 Sep 19;11(18):2923. doi: 10.3390/foods11182923 (PMC9498773; doi:10.3390/foods11182923)
Supplement: Supplementary file 1 [file foods-11-02923-s001.zip › foods-1894981-supplementary.pdf]

foods

MDPI

SUPPLEMENTARY MATERIAL

Supplemental Figure S1. Monthly mean temperature, precipitation sum and evapotranspiration during the (**a**) 2015, (**b**) 2016, (**c**) 2017 and (**d**) Walter-Lieth climate diagram

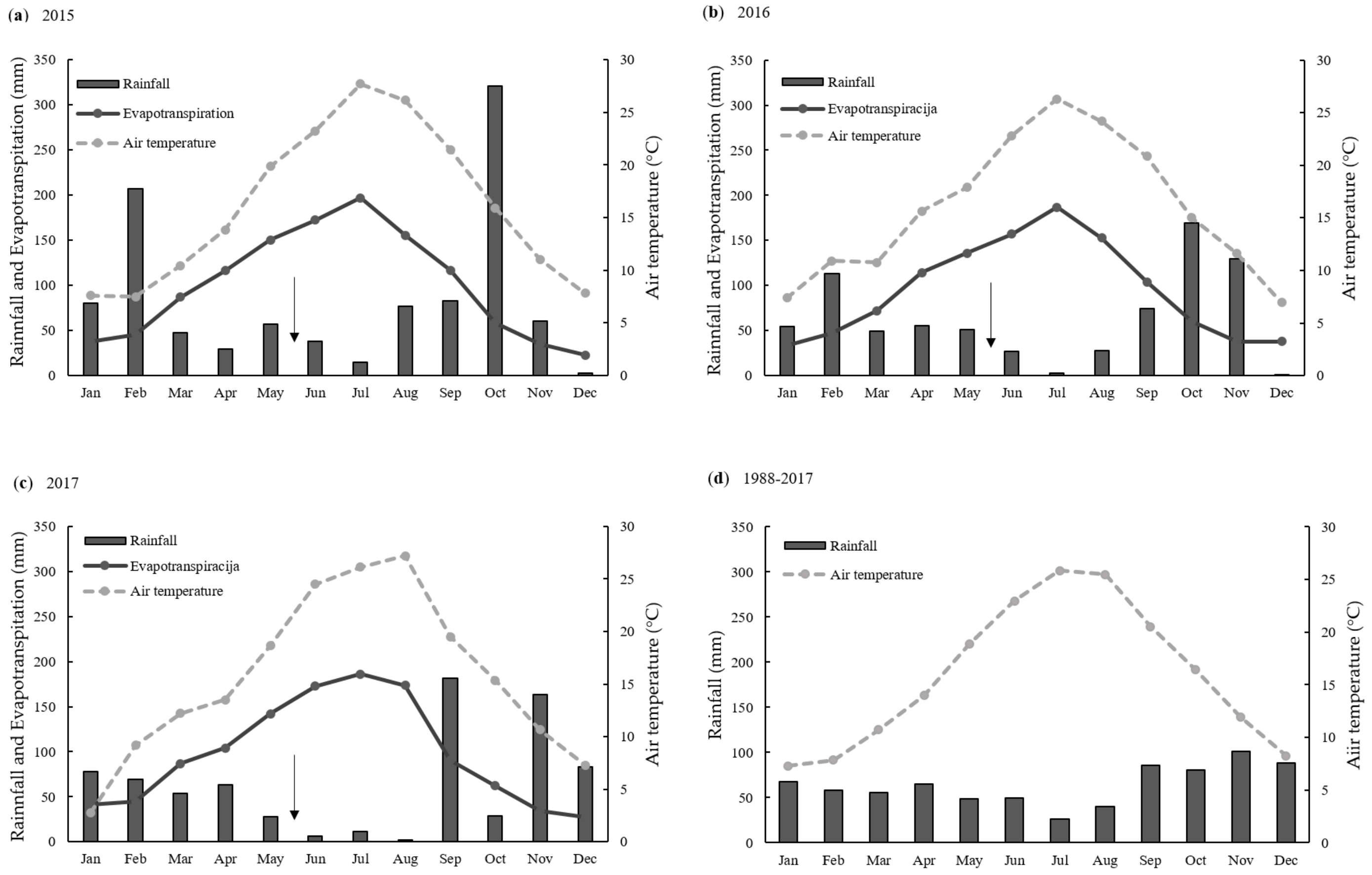

*Arrow indicates beginning of the irrigation period
